# Targeting the Extra-Cellular Matrix—Tumor Cell Crosstalk for Anti-Cancer Therapy: Emerging Alternatives to Integrin Inhibitors

**DOI:** 10.3389/fonc.2020.01231

**Published:** 2020-07-22

**Authors:** Girieca Lorusso, Curzio Rüegg, François Kuonen

**Affiliations:** ^1^Experimental and Translational Oncology, Department of Oncology Microbiology and Immunology, Faculty of Science and Medicine, University of Fribourg, Fribourg, Switzerland; ^2^Department of Dermatology and Venereology, Hôpital de Beaumont, Lausanne University Hospital Center, Lausanne, Switzerland

**Keywords:** extracellular matrix, tumor, progression, crosstalk, clinical perspectives

## Abstract

The extracellular matrix (ECM) is a complex network composed of a multitude of different macromolecules. ECM components typically provide a supportive structure to the tissue and engender positional information and crosstalk with neighboring cells in a dynamic reciprocal manner, thereby regulating tissue development and homeostasis. During tumor progression, tumor cells commonly modify and hijack the surrounding ECM to sustain anchorage-dependent growth and survival, guide migration, store pro-tumorigenic cell-derived molecules and present them to enhance receptor activation. Thereby, ECM potentially supports tumor progression at various steps from initiation, to local growth, invasion, and systemic dissemination and ECM-tumor cells interactions have long been considered promising targets for cancer therapy. Integrins represent key surface receptors for the tumor cell to sense and interact with the ECM. Yet, attempts to therapeutically impinge on these interactions using integrin inhibitors have failed to deliver anticipated results, and integrin inhibitors are still missing in the emerging arsenal of drugs for targeted therapies. This paradox situation should urge the field to reconsider the role of integrins in cancer and their targeting, but also to envisage alternative strategies. Here, we review the therapeutic targets implicated in tumor cell adhesion to the ECM, whose inhibitors are currently in clinical trials and may offer alternatives to integrin inhibition.

## Introduction: Targeting the ECM-tumor Cell Crosstalk

The extra-cellular matrix (ECM) is a dynamic niche continuously undergoing quantitative and qualitative remodeling by renewed synthesis and proteolytic modifications. During ECM remodeling, changes to its physical structure and organization occur, leading to a dysregulation in fiber composition, tissue architecture, and stiffness contributing to cancer progression and fibrosis (1). The cell can sense the surrounding ECM fibers by transmembrane surface molecules, such as integrins or other glycoproteins, acting as cellular mechano-chemical sensors. The relevance of the finely tuned integration and crosstalk between the ECM molecules, the cellular cytoskeleton, and the downstream signaling pathways, has been widely recognized and studied (2, 3). Their complex dynamic bi-directional interactions and mechano-transduction control have been associated to fundamental physiological processes such as branching tissues morphogenesis and angiogenesis during development and homeostasis. These interactions are also relevant to pathological conditions including cancer, from initial malignant transformation to the disruption of tissue polarity and promotion of invasiveness toward dissemination and metastasis development (4, 5). Integrins represent the key cell surface receptors for the cell to sense the ECM, triggering signaling pathways that determine cell fate and evolution toward a malignant phenotype and resistance to therapy (6, 7). Numerous experimental and preclinical studies conducted over the past decades highlighted the central role of integrins in affecting different steps of tumorigenesis, by controlling tumor cell adhesion, proliferation, migration, invasion, and survival (6). This made integrins appealing therapeutic targets leading to the development of integrin inhibitors and their clinical testing in cancer trials. Unfortunately and unexpectedly, integrin inhibitors failed to deliver any tangible therapeutic benefits for cancer patients (8–10). This failure may be due to the intrinsic complexity of integrin signaling that we still do not fully understand. But they also question the pharmacokinetic/pharmacodynamics properties of the integrin inhibitors developed, the integrin subunit and the associated biological process targeted, the preclinical models used as well as the design of the clinical trials performed (7, 8). Addressing those yet unanswered questions is likely to pave the road toward successful introduction of a novel generation of integrin inhibitors in clinical practice. In the meantime, long-ago discovered non-integrin ECM receptors as well as intra-cellular downstream effectors of the ECM-tumor cell crosstalk (signaling molecules) taking part in several key aspects of tumor progression, were largely neglected. Considering the clinical failure of integrin inhibitors, these ECM-tumor crosstalk targets are potential candidates that may be therapeutically exploited in alternative to integrin inhibitors. Here we review those currently tested in anti-cancer clinical trials, and portray their biology and activity in promoting tumor evolution.

## Non-integrin Tumor Cell Receptors to the ECM

### CD44

**Figure 1:**
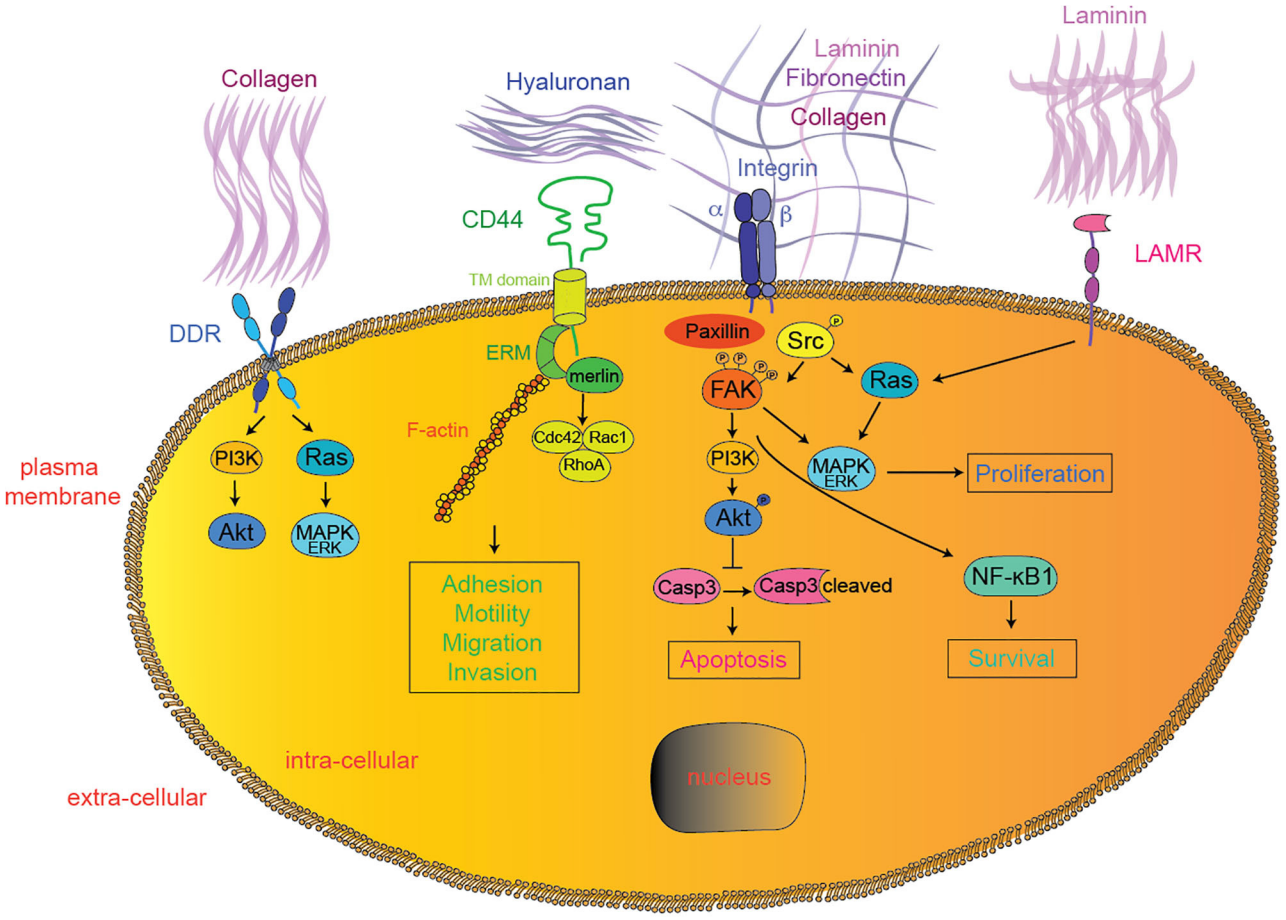
Extracellular matrix—tumor cell interactions. In addition to integrins, DDR, CD44, LAMRs, FAK, and SFK represent emerging therapeutic targets currently tested in clinical trials for solid tumors. Downstream effectors interactions were simplified for clarity reasons. DDR, discoidin domain receptor; LAMR, 36/67 kDa laminin receptors; FAK, focal adhesion kinase; PI3K, phosphoinositide-3-kinase; MAPK, mitogen-activated protein kinases; Casp3, caspase 3; NF-κB1, nuclear factor-kappa B1.

CD44 is a non-kinase transmembrane glycoprotein expressed in various cancer types (11). CD44 extracellular domain contains binding sites for various ECM proteins such as collagen, laminin, and fibronectin (12, 13), while hyaluronic acid (HA) produced both by tumor cells and tumor stroma is the main and most specific CD44 ligand (14, 15) (Figure 1). CD44 functions are modulated by both glycosylation and alternative splicing (16–18). Unlike the standard CD44 (CD44s), variant CD44 isoforms (CD44v) contain exons with specific post-translational modifications allowing binding of tumor-promoting cytokines like osteopontin (OPN), hepatocyte growth factor (HGF), vascular endothelial growth factor (VEGF), and basic fibroblast growth factor (bFGF) (19–23). Upon HA binding, CD44 proteins change conformation, oligomerize, and redistribute in glycolipid-enriched domains (GEMs) at the cell membrane (24, 25). There, activated CD44 preferentially interacts with activated receptor tyrosine kinases (RTKs) (26), various adaptor proteins such as ankyrin or the ERM (ezrin, radixin, and meosin), ultimately leading to cytoskeletal changes (spectrin, F-actin) (27, 28), Src family kinases (SFK) members accumulation (29), and activation of downstream pathways, such as Rho-GTPases (30–33), PI3K/AKT, or Ras/MAPK (34, 35) (Figure 1). Since the seminal discovery of their role in metastasis (36), CD44s and CD44v have been implicated in various steps of tumor progression. In particular, HA-induced CD44 conformational changes and subsequent cytoskeletal modifications promote tumor cell migration, invasion, and epithelial-to-mesenchymal transition (EMT) (27, 28, 30, 37–45). In glioma cells, HA-CD44 interactions were shown to occur specifically at the leading edge of migrating cells upon regulation by activated protein kinase C (PKC) (46). Upon HA binding, various proteases cleave CD44 allowing dynamic cytoskeletal changes, filopodia formation and ultimately CD44-mediated migration (47–50). Recently, non-catalytic MMP-9–mediated activation of CD44 was shown to promote tumor cell amoeboid migration (51). Since mesenchymal migration is based on integrin—ECM interactions, it is tempting to hypothesize that CD44 may support migration plasticity and escape to integrin inhibition (52–54). Further along tumor progression, circulating tumor cells (CTC) need to extravasate at distant organs. CD44 expressed on CTC was shown to interact with the HA coat produced by endothelial cells and initiate the process of tumor cell extravasation (55), particularly to the bone marrow, as shown in various tumor models through *in vitro* studies (56, 57). Importantly, both Cathepsin K, a potent collagenase typically expressed by osteoclasts during osteolysis, and MMP-9 were reported to be induced upon HA-mediated CD44 activation in prostate and breast cancer cells, suggesting their role in the colonization of metastatic osteolytic prostate and/or breast cancer cells (58–60). CD44 alternative splicing was reported to promote lung colonization by metastatic cancer cells (61). Recent studies implicated HA-CD44 interaction in tumor cell resistance to chemotherapy, by inducing multi-drug resistance 1 gene (MDR1) expression (62), ABC drug transporters (63), ankyrin-induced drug fluxes (62), and tumor cell survival pathways like ErbB2 signaling and PI3K/AKT pathway (64). Alternatively, HA-CD44 interactions may provide chemo-resistance through decreased apoptosis/cell death pathways by inducing anti-apoptotic proteins like inhibitors of the apoptosis family members (IAPs) (65–68), reducing pro-apoptotic proteins (69) or modulating autophagy (70).

Altogether, CD44 is involved at multiple steps of tumor progression and its inhibition appears as a promising alternative for tumor-ECM targeting therapies. Low molecular mass HA, soluble CD44, CD44 blocking antibodies, CD44 blocking peptides/aptamers, CD44-targeting sh/siRNA or silibinin (a plant-derived inhibitor of CD44 expression) have all been used successfully to interfere with CD44 function in preclinical models of solid tumor progression (Table 1). The CD44-blocking antibody RO5429083 was tested in a phase I, dose-escalation clinical study in metastatic or locally advanced, CD44-positive malignant solid tumors (NCT01358903) as well as in a phase I clinical study, alone or in combination with cytarabine, for acute myelogenous leukemia (NCT01641250). Alternatively, CD44 targeting may serve to specifically deliver cytotoxic drugs or radioisotopes to tumor cells. Bivatuzumab-mertansine, a CD44v6-specific targeting antibody linked to the cytotoxic drug mertansine, was tested in phase I dose-escalation clinical studies for CD44v6-positive recurrent or metastatic breast cancers (NCT02254031, NCT02254005) and advanced squamous cell carcinoma of the head and neck (NCT02254044, NCT02254018). The ^186^Re-labeled bivatuzumab was tested in phase I biodistribution studies for non-small cell lung cancers (NCT02204059) and adenocarcinoma of the breast (NCT02204046). Although preliminary, these results encourage further clinical assessment of CD44-targeting therapies, either alone or in combination.

**Table 1:** *In vivo* preclinical studies for solid tumors.

	**Molecule**	**Combination**	**Tumor model**	**Biological process**	**References**
Targeting CD44	Low molecular mass HA	–	Ovary, peripheral nerve	Tumor growth/metastasis	(71–73)
	soluble CD44	–	Melanoma, breast	Tumor growth	(74–76)
	CD44 blocking antibody	–	Breast, colon, pancreas, liver	Tumor growth, metastasis	(77–81)
	CD44v6 blocking antibody	–	Pancreas	Metastasis	(80, 82, 83)
	CD44 peptide	–	Melanoma, gastric	Tumor growth/metastasis	(81, 83–85)
	CD44v3 peptide	–	Glioblastoma	Tumor growth	(71–73, 84, 86)
	CD44v6 si/shRNA	–	Colon, gastric	Tumor growth	(82)
	CD44/Epcam aptamer		Ovary	Tumor growth	(82, 85)
	Silibinin	–	Prostate	Tumor growth	(86)
Targeting DDR	DDR1 blocking antibody	–	Breast	Tumor growth	(87)
	7rh (DDR1 inhibitor)	–	Gastric, pancreas	Tumor growth	(88, 89)
	WRG-28 (DDR2 inhibitor)	–	Breast	Metastasis	(90)
	Dasatinib (multikinase inhibitor)	–	Lung	Tumor growth	(91)
	Nilotinib (multikinase inhibitor)	–	Colon	Metastasis	(92)
	7rh (DDR1 inhibitor)	Dasatinib	Nasopharyngeal carcinoma	Tumor growth	(93)
	7rh (DDR1 inhibitor)	LY-411575 (Notch inhibitor)	Lung	Tumor growth	(91)
	DDR1-IN1 (DDR1 inhibitor)	Temzolomide/radiotherapy	Glioblastoma	Tumor growth	(94)
	Dasatinib (multikinase inhibitor)	JQ1 (BET inhibitor)	Lung	Tumor growth	(95)
	LAMR small molecule inhibitor	–	Breast	Metastasis	(96)
Targeting LAMR	LAMR^37^ blocking antibody	–	Fibrosarcoma	Metastasis	(97)
	OFA/iLRP-blocking antibody	–	Melanoma	Metastasis	(98–100)
	OFA/iLRP-based immunotherapy	–	Fibrosarcoma, sarcoma	Tumor growth/metastasis	(99, 100)
	FAK C-terminal domain	–	Fibroblasts, breast	Tumor growth/metastasis	(101, 102)
Targeting FAK	TAE-226	–	Glioma, ovary	Tumor growth	(103)
	VS-6062 (FAK/Pyk2 inhibitor)	–	Prostate, pancreas, melanoma, basal cell carcinoma	Tumor growth/metastasis	(104–107)
	VS-4718	–	Breast, ovary	Tumor growth/metastasis	(108, 109)
	VS-6063	–	Ovary	Tumor growth	(110)
	Compounds 14, Y15, Y11	–	Breast, pancreas, colon	Tumor growth	(111–114)
	Compounds C4, INT2-31, M13, R2 (FAK scaffold inhibitors)	–	Breast, pancreas, neuroblastoma, melanoma, colon	Tumor growth	(115–121)
	BI853520	–	Breast, mesothelioma	Tumor growth	(122, 123)
	NVP-TAE-226	–	Ewing sarcoma	Tumor growth/metastasis	(124)
	NVP-TAE-226	Docetaxel	Ovary	Tumor growth	(125)
	VS-6062 (FAK/Pyk2 inhibitor)	Sunitinib	Liver	Tumor growth	(126)
	VS-6062 (FAK/Pyk2 inhibitor)	Vemurafenib	Colon	Tumor growth	(127)
	Compound Y15	5-FU	Colon	Tumor growth	(113)
	Compound Y15	Gemcitabine	Pancreas	Tumor growth	(112, 128)
	Compound C4 (FAK scaffold inhibitor)	Temzolomide	Glioblastoma	Tumor growth	(128)
		Doxorubicin	Breast	Tumor growth	(115)
	Compound R2 (FAK scaffold inhibitor)	Doxorubicin, 5-FU	Colon	Tumor growth	(121)
	PF5735228	WZ811 (CXCR4 inhibitor)	Lung	Tumor growth	(129)
	VS-4718	HDAC inhibitors	Lung, Esophagus	Tumor growth	(130)
	VS-4718	PD-1 antagonist, T cell immunotherapy	Pancreas	Tumor growth	(131)
	VS-6063	Docetaxel	Prostate	Tumor growth	(132)
	FAKsi nanoparticles	Paclitaxel nanoparticles	Ovary	Tumor growth	(133)
	Bosutinib (multikinase inhibitor)	–	Neuroblastoma, thyroid, prostate, pancreas, colon	Tumor growth/metastasis	(134–139)
Targeting SFK	Dasatinib (multikinase inhibitor)	–	Prostate, pancreas, colon	Tumor growth/metastasis	(140–142)
	Saracatinib (multikinase inhibitor)	–	Pancreas, prostate, head and neck, liver, gastric, biliary, sarcoma, colon, skin	Tumor growth/metastasis	(143–153)
	Ponatinib (multikinase inhibitor)	–	Glioblastoma, neuroblastoma, endometrial, gastric, breast, lung, bladder, colon, rhabdomyosarcoma, GIST	Tumor growth	(154–159)
	Vandetanib (multikinase inhibitor)	–	Breast, thyroid, glioblastoma, lung, liver, prostate, head and neck, vulva, ovary, gastric, pancreas, kidneys, colon	Tumor growth/metastasis	(160–185)
	Dasatinib (multikinase inhibitor)	Cetuximab	Colon	Tumor growth	(186)
	Dasatinib (multikinase inhibitor) Saracatinib (multikinase inhibitor)	Erlotinib/gemcitabine	Pancreas	Tumor growth	(187)
		Axitinib/erlotinib	Colon	Tumor growth	(188)
		Trastuzumab	Breast	Tumor growth	(189)
		Trametinib	NSCLC	Tumor growth	(190)
		Bevacizumab	Glioma	Tumor growth	(191)
		Rapamycin	Liver	Tumor growth	(192)
		Paclitaxel	Breast, ovary	Tumor growth	(193, 194)
		Cisplatin	Bladder	Tumor growth	(195)
		Oxaliplatin	Colon	Tumor growth	(194, 196)
		Gemcitabine	Urothelial	Tumor growth	(197)
		Vincristine	Breast	Metastasis	(198)
		MCL-1 inhibitor	Breast	Tumor growth	(199)
		CYT997	Prostate	Tumor growth/metastasis	(200)
		Caffeic acid phenetyl	Glioma	Tumor growth	(201)
		Dendritic cell vaccine	Breast	Tumor growth/metastasis	(202)
		Anti-CTLA-4	Head and neck	Tumor growth	(203)
		Cetuximab	NSCLC	Tumor growth	(204)
	Saracatinib (multikinase inhibitor) Vandetanib (multikinase inhibitor)	Cabozantinib	Schwannoma	Tumor growth	(205)
		Capivasertib	Head and neck	Tumor growth	(206)
		Trastuzumab	Breast, gastric	Tumor growth	(207, 208)
		Anastrozole	Breast	Tumor growth	(209)
		Fulvestrant	Ovary, breast	Tumor growth	(210, 211)
		5-FU	Gastric	Tumor growth	(212)
		Celecoxib	Osteosarcoma	Tumor growth	(213)
	Vandetanib (multikinase inhibitor)	Tamoxifen	Breast	Tumor growth	(214)
		Paclitaxel	Ovary, colon	Tumor growth/metastasis	(215, 216)
		Cisplatin	Neuroblastoma	Tumor growth	(217)
		Oxiplatin	Colon	Tumor growth	(218)
		Temozolomide	NSCLC, glioblastoma	Tumor growth	(219–221)
		Radiotherapy	Head and neck, lung	Tumor growth	(222)
		Radiotherapy/gemcitabine	Pancreas	Tumor growth	(223)
		Radiotherapy/irinotecan	Colon	Tumor growth	(224)
		Radiotherapy/cisplatin	Head and neck	Tumor growth	(225)
		L19m-TNFalpha	Esophagus	Tumor growth	(226)

### Discoidin Domain Receptors (DDR)

DDR1 and DDR2 belong to the family of the transmembrane receptor tyrosine kinase (RTK) with an extracellular discoidin domain binding to collagen in its native triple-helical conformation (227, 228) (Figure 1). DDR1 and DDR2 bind to various collagen isoforms with different affinities. DDR1 typically binds to collagens I-VI and VIII, while DDR2 preferentially binds to collagens I-III and X (228–231). Upon collagen binding, DDRs cluster and get activated through auto-phosphorylation at multiple tyrosine residues within the cytosolic part of the protein (232, 233), leading to the recruitment of adaptor or signaling proteins like ShcA, SHP-2, SFKs, the proline-rich tyrosine kinase 2 (Pyk2), and the non-muscle myosin heavy chain (NMHC) IIA (234, 235). In cancer cells, DDR activation was reported to induce Ras/MAPK (236), PI3K/AKT (236), Notch (237), NF-κB (238), PKCα/JAK/Stat (239), and p130CS/JNK pathways (234), thereby participating in various steps of tumor progression (Figure 1). Both DDR1 and DDR2 were shown to promote tumor cell proliferation, survival (236, 238, 240, 241), and migration (242–245). Interestingly, EMT was reported to rely on the switch from DDR1 (epithelial) to DDR2 (mesenchymal) expression (246), although various reports implicate both DDR1 and DDR2 in EMT-mediated tumor cell invasion (234, 247). More recently, DDRs were implicated in the late stages of metastatic tumor progression (244, 248). Typically, DDR1 drives site-specific metastasis of lung cancer cells to bone (248). Additionally, the collagen-dependent interaction between Transmembrane 4 L6 Family Member 1 (TM4SF1) and DDR1 regulates dormancy vs. growth at the metastatic site (239). Finally, both DDR1 and DDR2 promote resistance to radio- and chemo-therapy in various preclinical models (94, 236–238, 249). However, despite these converging evidences implicating DDRs in tumor progression, one should consider that DDR-mediated effects are highly versatile and cell-dependent. For example, DDR1 was shown to either support or prevent integrin α2β1-mediated cell migration in different experimental models (234, 250, 251). Moreover, the dynamic regulation of DDR expression during tumor progression will determine the consequences of DDR inhibition (231). Thus, the complex regulation of DDR activity in tumor cells may stand for the controversy concerning their contribution to cancer progression (243, 248, 252–254) and affect the potential efficacy of DDR targeting in cancer. Still, the recent identification of activating mutations in the cytoplasmic signaling portions of DDR affecting intracellular signaling (240, 255–257) opens new perspectives in the identification of patients who might benefit the most from DDR inhibition.

DDR1 and DDR2 kinases are efficiently inhibited by multikinase inhibitors like ponatinib, imatinib, dasatinib, and nilotinib (258). Dasatinib, nilotinib, a DDR1 blocking antibody, the selective DDR1 inhibitors 7rh and DDR1-IN-1 and the selective allosteric DDR2 inhibitor WRG-28 were shown to efficiently prevent DDR-mediated tumor progression in preclinical models (Table 1). Driven by these encouraging results, dasatinib was tested in a phase II clinical trial for patients with advanced non-small cell lung cancers harboring a DDR2 mutation (NCT01514864). Unfortunately, it was abandoned because of lack of efficacy and slow enrollment. Currently, nilotinib is being assessed in a phase II clinical trial for malignant locally advanced or metastatic solid neoplasms presenting DDR1 or DDR2 mutations (NCT02029001). Importantly, non-canonical activation of DDR1 was shown to promote metastasis through tyrosine kinase-independent signaling in preclinical models (239), warranting cautious assessment of RTK inhibitors to target DDR. Further efforts should aim at the development of specific DDR1 and DDR2 inhibitors targeting canonical and non-canonical activation routes, the identification of the patients who may benefit the most from DDR inhibition and their use in combination therapies.

### 36/67 kDa Laminin Receptors (LAMR)

The 67 kDa (LAMR^67^) laminin receptor was first identified as a receptor for laminin 1 (259–261) (Figure 1). It is currently hypothesized that LAMR^67^ arises from post-translational modifications of the precursor 37 kDa laminin receptor (LAMR^37^), although the precise mechanisms (like sumoylation) are still to be resolved (262–264). LAMRs harbor multiple cellular localizations, as assessed by the wide range of cellular processes they are implicated in: ribosomal biogenesis (265), protein translation (266–268), pre-rRNA processing (269), cellular adhesion and migration (267, 270), invasion (271), cellular proliferation (272, 273), cytoskeletal modulation (267, 274), and chromatin and histone modifications (275). Both LAMR^37^ and LAMR^67^ were identified at the cell membrane where they potentially bind to laminins, associate with integrins (276, 277) and get phosphorylated (278, 279). Although the downstream signaling mechanisms are still unelucidated, various authors reported modifications of Ras/MAPK and JNK/p38 signaling upon laminin-binding to LAMRs (280), possibly through interactions with FAK and paxillin (267, 281) (Figure 1). Given their various implications in cellular regulation, it is not surprising to find elevated LAMR expression in various cancers (282–288) and their involvement in tumor cell growth, migration, invasion, and aggressiveness (266, 282, 289). Importantly, laminin 1—LAMR interaction was shown to be implicated in tumor cell adhesion (271, 290) and invasion (291, 292) and LAMR down-regulation was shown to promote tumor cell apoptosis (293–296). Whether this is mediated by laminin 1-dependent activation of LAMR remains unknown. Recent data suggest that LAMR interaction with FAK may depend on laminin 1—LAMR interaction and promote Ras/MAPK and/or PI3K/AKT-mediated survival (297, 298). However, LAMR was found to promote tumor progression through various laminin 1-independent manners, such as regulation of telomerases (299), reviewed in (300).

Despite various emerging strategies aimed to target LAMR (300), *in vivo* preclinical studies assessing the feasibility and efficiency of targeting LAMR are still scant. Both a LAMR^37^ blocking antibody and a small molecule inhibitor preventing laminin-LAMR interaction were shown to impede metastatic progression (Table 1). The green tea-derived epigallocatechin-3-gallate (EGCG) is a small molecule affecting a large number of cellular targets, including LAMR^67^ (301) and LAMR^37^ (302). EGCG is currently assessed in a phase I study for chemopreventive effect in patients with curative-intent resections of colorectal cancer (NCT02891538). Interestingly, the immunogenic LAMR tumor-associated antigen, referred as oncofoetal antigen immature laminin receptor protein (OFA-iLRP), has been successfully used as a tumor antigen for vaccine-based therapies in preclinical studies (Table 1). Cellular immunotherapy using autologous dendritic cell loaded with OFA-iLRP was tested in a phase I-II clinical study for metastatic breast cancers (NCT00879489). Altogether, LAMR targeting appears promising for cancer therapy, although major efforts should aim at the development of specific inhibitors and acquisition of stronger preclinical data prior to further clinical trial.

## Downstream Effectors of Integrin-Mediated Tumor Cell Adhesion to the ECM

### Focal Adhesion Kinase (FAK)

Focal adhesion kinase (FAK) is a cytoplasmic non-receptor protein tyrosine kinase. It is an important cell signaling hub highly phosphorylated upon integrin activation, and has long been recognized as promoting cancer cell migration, proliferation, and survival/chemoresistance through downstream activation of Rho-GEF, talin, cortactin, SFKs, PI3K/AKT, Ras/MAPK, or NF-κB pathways (303, 304) (Figure 1). More recent studies have described that besides its classical localization at the plasma membrane of tumor cells, FAK can also translocate to the nucleus and act as a transcription factor driving the expression of cytokines and chemokines favoring tumor immune evasion, independently of integrin signaling (305). In pancreatic cancer, FAK inhibition increases the immune infiltrate within the tumor environment, thereby sensitizing tumors to immune-checkpoint blockade (306). In addition, FAK inhibition also affect stromal cells. By targeting carcinoma-associated endothelial cells, FAK inhibition enhances vascular permeability, drug delivery, and overcomes chemo-resistance to DNA-damaging agents (307). Altogether, these data largely support the potential for therapeutic benefits of FAK inhibitors, used alone or in combination therapies, in the arsenal of anti-cancer strategies, illustrated by their success in various preclinical models (Table 1). FAK inhibition mostly relies on small molecule inhibitors working through various mechanisms: ATP competitive kinase inhibition (TAE-226, VS-4718, VS-6062, VS-6063, GSK-2256098, PF-573228), FAK scaffold inhibition (compounds 14, Y11, Y15, C4, INT2-31, M13, R2), or more recently ATP competitive non-kinase inhibition (BI853520) (Table 1). In combination, FAK inhibition was reported to improve the efficacy of chemotherapeutic agents (docetaxel, paclitaxel, temzolomide, 5-FU, gemcitabine, doxorubicin), targeted therapies (EGFR inhibitor, Src inhibitor, sunitinib, BRAF inhibitor, CXCR4 inhibitor, HDAC inhibitor), or immunotherapy (PD1 antagonists, T cell immunotherapy) (Table 1). Acceptable safety profiles were obtained in phase I clinical trials for VS-6062 (104, 308), GSK-2256098 (309–311), VS-6063 (312, 313), VS-4718 and BI853520 (314–316), with VS-6062, GSK-2256098, and VS-6063 showing stabilization of disease in patients with various advanced solid tumors. Both GSK-2256098, in combination with trametinib, and VS-6063, however, failed to show efficacy in phase II clinical trials for pancreatic adenocarcinoma and malignant mesothelioma, respectively [NCT02428270, (317)]. This unexpected failure may have been prevented by the stratification of the patients based on FAK amplification/activity in order to select for the best responders. VS-6063 is currently tested in multiple clinical trials: (i) a phase II clinical trial in a pre-operative setting for malignant mesothelioma (NCT02004028); (ii) a phase II clinical trial in association with the PD-1 inhibitor pembrolizumab for advanced solid tumors (NCT02758587, NCT03727880); (iii) a phase I clinical trial in association with the RAF/MEK inhibitor RO5126766 for advanced solid tumors (NCT03875820); (iv) a phase I clinical trial in association with the anti-PDL1 antibody avelumab for epithelial ovarian cancer (NCT02943317); (v) a phase I clinical trial in association with pembrolizumab and gemcitabine for advanced solid tumors (NCT02546531). The results of these ongoing clinical trials will be decisive to shape the future development of FAK inhibitors in clinical practice.

### Src Family Kinases (SFK)

The SFK, composed of c-Src, Fyn, Yes, Lck, Lyn, Hck, Fgr, and Blk, are cytoplasmic non-receptor protein tyrosine kinases. Their prominent functions are mediated by their SH2 and SH3 domains interacting with various RTKs (such as EGF-R, HER2, IGF-R, HGF-R, and PDGF-R), thereby participating in integration and regulation of RTK signaling. But SFK also participate in ECM-mediated signaling. Through phosphorylation of FAK, SFK activation stabilizes focal adhesion complexes enhancing cell adhesion to the ECM (318) (Figure 1). Altogether, SFK are implicated in many steps of tumorigenesis, including proliferation, migration, invasion, survival in the circulation and at distant metastatic sites (319–324), achieved through modulation of various downstream effectors as PI3K/AKT, Ras/MAPK, or Stat3 (325, 326). Additionally, SFK activation confers therapeutic resistance to targeted RTK therapies (e.g., Trastuzumab/Herceptin for HER2), to hormone-receptor endocrine therapies (e.g., Tamoxifen for Estrogen Receptor), as well as to traditional chemo- and radiotherapies (327). Given their central role in tumor cell signaling and pleiotropic functions in cancer, SFK represent a promising target for anti-cancer therapies. SFK are currently most efficiently targeted using non-specific ATP-competitive multikinase inhibitors, such as dasatinib, bosutinib, saracatinib, ponatinib, and vandetanib, targeting many different tyrosine kinases (such as BCR-ABL, Kit, PDGFR, EGFR, RET, VEGFR) in addition to SFK members (328). With the exception of vandetanib, approved for the treatment of thyroid medullary carcinoma, dasatinib, ponatinib, and bosetanib have been approved by the FDA for hematological malignancies only, based on their BCR/Abl inhibitory capacity (328). *In vivo* preclinical data, however, suggest their potential efficacy in solid tumors as well, alone or in combination, although not necessarily through SFK inhibition (Table 1). Up to date, the results of phase II clinical trials with SKF inhibitors in monotherapy have been disappointing, as they showed only modest or no efficacy (326, 329). Such failure may be largely attributed to the current lack of biomarkers for the identification patients with aberrant SFK, the lack of specificity of SFK inhibitors, and the sometimes opposing effects of SFK members at various steps of tumor progression (330, 331). The interpretation of the numerous ongoing clinical trials (http://www.clinicaltrials.gov/) as well as the design of future successful clinical trials testing SFK inhibitors for solid tumors will largely depend on our capacity to overcome these important issues.

## Conclusion

Despite huge expectations based on preclinical studies, integrin inhibitors failed to deliver anticipated results and have not entered the clinical practice yet. Understanding and surmounting the pitfalls of integrin inhibition will be crucial to further sustain the targeting of tumor cell–ECM interactions as an anticancer strategy. Yet, other long-time discovered molecules at the interface between tumor cell and ECM as CD44, DDR, LAMR, FAK, and SFK, are emerging as alternative therapeutic targets in clinical trials. Alike integrin inhibitors, their therapeutic relevance will depend on the specificity and pharmacokinetic/dynamic properties of the inhibitors developed, on the adequacy of the preclinical models used for validation, on the biological process targeted, on the biomarkers used for the identification of best responders and on the combination strategies applied in clinical trials. Importantly, our growing knowledge of the biology of ECM—tumor cell interactions will be instrumental in overcoming these important pitfalls and extend the arsenal of clinically valuable inhibitors targeting the ECM—tumor cells crosstalk in the near future.

## Author Contributions

GL wrote the review and edited the manuscript. CR edited the manuscript. FK planned the outline, wrote the review, and edited the manuscript. All authors read and approved the submitted version of the manuscript.

## Conflict of Interest

The authors declare that the research was conducted in the absence of any commercial or financial relationships that could be construed as a potential conflict of interest.
